# Glucose Responsive Coacervate Protocells from Microfluidics for Diabetic Wound Healing

**DOI:** 10.1002/advs.202400712

**Published:** 2024-05-20

**Authors:** Chong Wang, Xinyuan Yang, Qiao Wang, Linyi Zhang, Luoran Shang

**Affiliations:** ^1^ Shanghai Xuhui Central Hospital Zhongshan‐Xuhui Hospital and the Shanghai Key Laboratory of Medical Epigenetics the International Co‐Laboratory of Medical Epigenetics and Metabolism (Ministry of Science and Technology) Institutes of Biomedical Sciences Fudan University Shanghai 200030 China

**Keywords:** coacervates, diabetic wound healing, glucose responsiveness, microcarriers, microfluidics, protocells

## Abstract

The hyperglycemic pathophysiological environment in diabetic wounds is a major obstacle that impedes the healing process. Glucose‐responsive wound healing materials are a promising approach to address this challenge. In this study, complex coacervate‐based protocells are introduced for diabetic wound healing. By employing a microfluidic chip with an external mechanical vibrator, uniform coacervate microdroplets are generated via electrostatic interactions between diethylaminoethyl‐dextran and double‐stranded DNA. The spontaneous assembly of a phospholipid membrane on the droplet surface enhances its biocompatibility. Glucose oxidase and copper peroxide nanodots are integrated into microdroplets, enabling a glucose‐responsive cascade that produces hydroxyl radicals as antibacterial agents. These features contribute to efficient antibacterial activity and wound healing in diabetic mice. The present protocells facilitate intelligent wound management, and the design of cascade catalytic coacervates can contribute to the development of various smart vehicles for drug delivery.

## Graphical abstract

This paper introduces a type of coacervate microdroplet‐based protocells for diabetic wound healing. These protocells, formed through electrostatic interaction, have a spontaneously assembled phospholipid membrane for enhanced biocompatibility. By integrating with glucose oxidase and copper peroxide nanodots, the protocells exhibit a glucose‐responsive cascade reaction, which produces antibacterial hydroxyl radicals. These protocells demonstrate promising results in diabetic mice, which hints at potential applications in smart drug delivery vehicles.

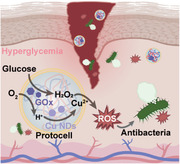

## 1 Introduction

Diabetic wound healing is a complex process involving several essential stages that work collectively for effective healing and tissue regeneration.^[^ ^1^, ^2^ ^]^ Various wound‐management materials have been developed, including hydrogel dressings and microparticles.^[^ ^3^, ^4^, ^5^ ^]^ By delivering bioactive therapeutic agents, such as antibiotics and growth factors, to the wound site, these materials can exert antimicrobial, anti‐inflammatory, immune‐modulatory, or angiogenic properties and help create a favorable microenvironment for the healing process.^[^ ^6^, ^7^, ^8^, ^9^ ^]^ However, a large proportion of these materials rely on the passive release of bioactive agents, which lack the ability to facilitate smart wound management. Although environmentally responsive hydrogels have been explored as delivery systems for wound healing, their complex synthetic processes restrict their practical application potential. Thus, novel delivery carriers that are sensitive to environmental stimuli in diabetic wounds are highly anticipated.

In this study, we propose complex coacervate‐based protocells as microcarriers for diabetic wound healing. Protocells are minimized cell models that inherit some of the basic structural and functional features of cells. Complex coacervates, typically formed through the liquid‐liquid phase separation (LLPS) of charged molecules, have been established as plausible protocells because of such formation mechanisms.^[^ ^10^, ^11^, ^12^ ^]^ Additionally, the coacervate droplets provide a mild compartment to selectively encapsulate biomolecules while preventing denaturation.^[^ ^13^ ^]^ These properties allow the coacervates to undergo biochemical reactions in response to specific environmental signals. Although many studies have focused on reproducing and examining cell‐like behaviors in coacervate‐based protocells, their use as intelligent drug‐delivery vehicles has been underestimated.^[^ ^13^, ^14^ ^]^ One of the major challenges is that coacervated microdroplets generated using conventional methods suffer from polydispersity and instability, which limits their biomedical applications.^[^ ^15^ ^]^ Therefore, methods for the fabrication of uniform and stable coacervate protocells should be investigated.^[^ ^16^, ^17^, ^18^ ^]^

**Figure 1:**
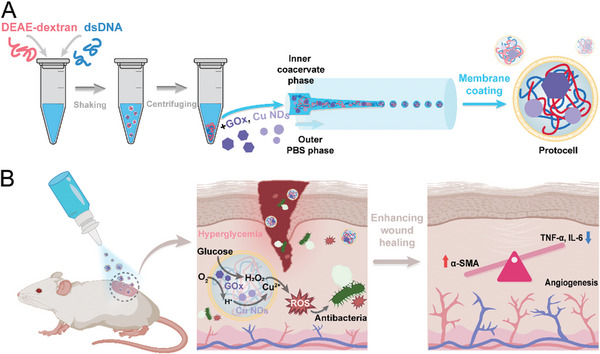
Schematic of the generation of GOx and Cu NDs‐loaded coacervate protocells and the working principle of promoting diabetic wound healing. A) The coacervate‐based protocells are generated by microfluidics, where a coacervate dense phase of DEAE‐dextran/dsDNA containing GOx and Cu NDs are emulsified in a microfluidic device and dispersed in a continuous PBS solution. After that, a phospholipid layer is spontaneously assembled onto the monodispersed DEAE‐dextran/dsDNA coacervate microdroplets. B) The coacervate microdroplet‐based protocells can generate ROS when glucose is present, thus showing antibacterial effects to promote diabetic wound healing.

Herein, we present a microfluidic approach for generating coacervate protocells with glucose‐responsive abilities for diabetic wound healing, as depicted in **Figure** **1**. Coacervate microdroplets consisting of diethyl‐aminoethyl‐dextran (DEAE‐dextran) and double‐stranded DNA (dsDNA) were prepared using a microfluidic device coupled with mechanical vibration. A phospholipid membrane was spontaneously assembled on the surface of the preformed microdroplets through electrostatic interactions to enhance their biocompatibility. Glucose oxidase (GOx) and copper peroxide nanodots (Cu NDs) were integrated within the coacervate droplets owing to their affinity partitioning properties.^[^ ^19^ ^]^ Coacervate droplets can leverage a hyperglycemic environment and initiate a reaction cascade to generate hydroxyl radicals, a type of reactive oxygen species (ROS). Therefore, we conducted in vitro and in vivo experiments to demonstrate that coacervate‐based protocells exhibit excellent biocompatibility, efficient antibacterial properties, and a remarkable ability to promote wound healing in a diabetic mouse model. These coacervate microcarriers offer smart and continuous management for promoting wound repair, and we believe that the design of such therapeutic protocells will benefit related biomedical fields.

## 2 Results and Discussion

### 2.1 Construction and Characterization of Phospholipid Membrane‐Enclosed Coacervates

**Figure 2:**
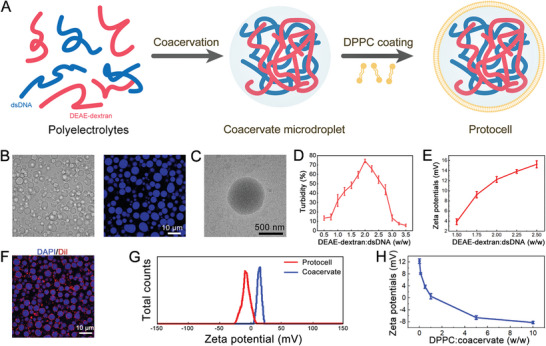
Construction and characterization of DPPC membrane‐coated coacervate droplets. A) The schematic of the formation of DEAE‐dextran/dsDNA coacervate microdroplets and the subsequent phospholipid coating. B) Bright‐field and fluorescence microscopy images of DAPI‐stained DEAE‐dextran/dsDNA coacervate microdroplets. C) TEM image of DEAE‐dextran/dsDNA coacervate microdroplets. D) Turbidity of DNA/DEAE‐dextran coacervate solution at different polyelectrolyte concentrations. Data are shown as mean ± SD (*n* = 5). E) Zeta potentials of DEAE‐dextran/dsDNA coacervate microdroplets produced at different polyelectrolyte weight ratios. Data are shown as mean ± SD (*n* = 5). F) Fluorescence microscopy image of DPPC membrane‐coated DEAE‐dextran/dsDNA coacervate microdroplets (stained with dye DAPI and Dil). G) Zeta potentials of coacervate microdroplets before and after adding 5 wt% DPPC into the solutions. H) Zeta potentials of protocells prepared with different relative contents of DPPC. Data are shown as mean ± SD (*n* = 5).

Before exploring microfluidics, we first studied the formation of phospholipid membrane‐enclosed coacervate droplets in a bulk solution, as shown in **Figure** **2**A. Aqueous solutions of dsDNA and DEAE‐dextran were directly mixed, resulting in the formation of a turbid suspension containing coacervate microdroplets. The discrete spherical droplets were observed under bright‐field and fluorescence microscopy, displaying homogeneous blue fluorescence when stained with DAPI staining solution, as depicted in Figure 2B. The transmission electron microscopy (TEM) image in Figure 2C further confirms the generation of DEAE‐dextran/dsDNA coacervate microdroplets. Additionally, varying polyelectrolyte weight ratios led to different turbidity levels and surface potentials, as shown in Figure 2D,E. To ensure the generation of a sufficient number of coacervate droplets, we opted for a 2:1 weight ratio of DEAE‐dextran to dsDNA for the subsequent experiment.

Next, DEAE‐dextran/dsDNA coacervate microdroplets with +12.3 ± 0.6 mV zeta potential (prepared at 2:1 DEAE‐dextran to dsDNA weight ratio) were utilized. 1,2‐Dipalmitoyl‐sn‐glycero‐3‐phosphocholine (DPPC), a two‐chain zwitterionic phospholipid, was assembled on the surface of the microdroplets. The successful coating of DPPC was demonstrated through confocal microscopy imaging, revealing a red fluorescence outer shell upon staining with Dil, as depicted in Figure 2F. Additionally, the coating of the DPPC membrane resulted in a decrease of the zeta potential to approximately −8.3 ± 0.91 mV at 5 wt% DPPC content (Figure 2G). With the increase of DPPC:coacervate ratio, the zeta potential of DPPC‐coated coacervate microdroplets decreased from +12.3 to −8.2 mV, as shown in Figure 2H. This can be attributed to the electrostatically mediated adsorption of DPPC onto the droplet surface. In the following text, we use the term “protocells” to refer to the coacervate microdroplets coated with DPPC. The above results validated that coacervate microdroplets and protocells can be successfully prepared by mixing. However, the products were not uniform in size, which limits their applications as microcarriers for delivering therapeutic agents.

### 2.2 Microfluidic Generation of Coacervate Protocells

**Figure 3:**
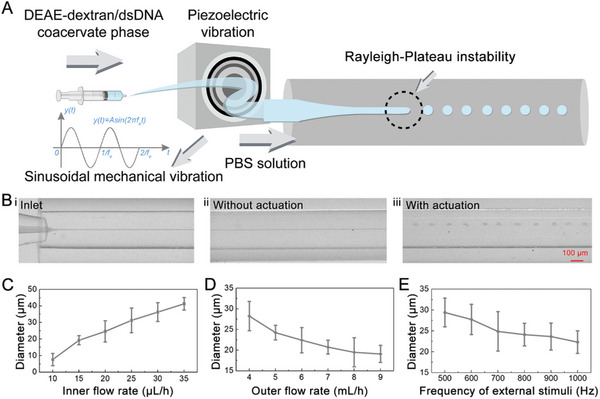
Fabrication of DEAE‐dextran/dsDNA coacervate microdroplets by microfluidics. A) Schematic of the generation of coacervate microdroplets by microfluidics. B‐i) The jet formation in the microfluidic device; (ii, iii) The downstream of the jet (i) before and (ii) after exerting mechanical actuation. C) Plot of the diameter of the coacervate microdroplets as a function of the inner phase flow rate. The outer phase flow rate was 5 mL h^−1^, and the actuation frequency was 800 Hz. Data are shown as mean ± SD (*n* = 100). D) Plot of the diameter of the coacervate microdroplets as a function of the outer phase flow rate. The inner phase flow rate was 20 µL h^−1^, and the actuation frequency was 800 Hz. Data are shown as mean ± SD (*n* = 100). E) Plot of the diameter of the coacervate microdroplets as a function of the frequency of mechanical actuation. The inner and outer phase flow rate was 20 µL h^−1^ and 5 mL h^−1^, respectively. Data are shown as mean ± SD (*n* = 100).

To reduce the heterogeneity of the generated protocells, we employed microfluidics to generate protocells with uniform size. This characteristic is advantageous for utilizing the protocells as microcarriers for delivering therapeutic agents. In this method, a dense DEAE‐dextran/dsDNA coacervate phase was first separated from the coacervate solution (formed by mixing) by centrifugation. Subsequently, the isolated coacervate phase was introduced into an axisymmetric flow‐focusing capillary microfluidic device, which served as the inner phase fluid. A phosphate‐buffered saline (PBS) solution containing DPPC served as the outer phase fluid, as shown in **Figure** **3**A. Due to the ultra‐low interfacial tension between the inner and outer phases, it is difficult to generate droplets by conventional passive method.^[^ ^20^ ^]^ Instead, a stable jet formed in the microfluidic channel all the way to downstream (Figure 3Bi,ii). To overcome this, we introduced mechanical perturbations to the inner phase fluid, which caused pressure fluctuation and forced the breakup of the jet to form protocell droplets.^[^ ^21^, ^22^ ^]^ The protocell droplets generated through such active microfluidics exhibited better monodispersity compared to those generated by mixing (Figure S1, Supporting Information). Additionally, the size of the droplets was contingent upon the inner and outer flow rates and the vibration frequency. Through this, it can be controlled within a certain range, as shown in Figure 3C–E. These results confirmed that the microfluidic approach offered a means to produce protocells with enhanced uniformity and size controllability.

### 2.3 Cascade Reaction Capacity of the Protocells

**Figure 4:**
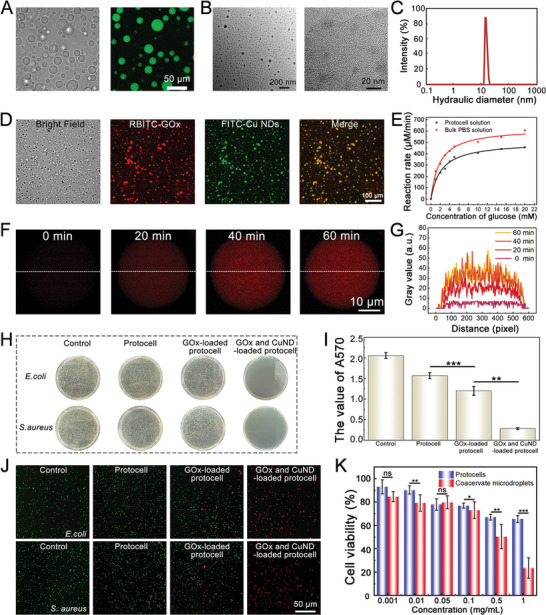
Cascade reaction capacity, antibacterial ability, and biocompatibility of the protocells. A) Bright‐field and fluorescence microscopy images demonstrating the affinity partitioning behavior of FITC‐GOx into the coacervate phase. B) TEM images at different magnifications demonstrating the successful synthesis of Cu NDs. C) Hydraulic diameter of Cu NDs. D) Bright‐field and fluorescence microscopy images showing the co‐encapsulation of FITC‐Cu NDs and RBITC‐GOx into the protocells. E) Michaelis−Menten plots of GOx as a function of glucose concentration in the bulk PBS solution and protocells. F) A series of fluorescence images of a single GOx‐loaded protocell showing the increase in red fluorescence in 60 min. G) Corresponding fluorescence intensity (gray values) profiles of the protocell displayed in F. H) Images of *E. coli* and *S. aureus* bacterial colonies on the culture plate after different treatments with the presence of 5 × 10^−3^ m glucose. I) Measurement of absorption of 570 nm of stained biofilm in each group. Data are shown as mean ± SD (*n* = 5). One‐way ANOVA and Tukey's test were performed. J) Fluorescence live/dead staining images of *E. coli* and *S. aureus* after coculturing with different solutions. K) Plot of cell viability against the concentration of uncoated and DPPC membrane‐coated coacervate droplets. Data are shown as mean ± SD (*n* = 5). Two‐tailed unpaired Student's t‐test was performed for the comparisons between the two groups.

We then investigated the properties of the coacervate‐based protocells, including their cascade catalytic activity, anti‐bacterial performance, and biocompatibility. In these sections (Sections 2.3–2.5), we conducted equivalent tests using protocells generated through rapid mixing. We incorporated glucose oxidase (GOx) and copper peroxide nanodots (Cu NDs) into the DPPC‐coated coacervates. As shown in **Figure** **4**A, GOx exhibits an affinity for the dense coacervate phase, with an affinity coefficient of approximately 74.86 (Figure S2, Supporting Information). Cu NDs, synthesized following previously reported protocols, had a small size range of approximately 10–20 nm (Figure 4B,C). The XRD spectrum suggests the presence of an amorphous structure or less crystallinity of the material (Figure S3A, Supporting Information). Meanwhile, the XPS spectra offer insights into the surface chemistry of Cu NDs (Figure S3B–D, Supporting Information). Similar to GOx, these Cu NDs can also be encapsulated within the coacervate microdroplets. Based on this, we successfully coencapsulated RBITC‐GOx and FITC‐Cu NDs within the coacervate microdroplets, as validated in Figure 4D.

Accommodating GOx and Cu NDs within the protocells allowed for a two‐step catalytic reaction. We started with analyzing the first‐step reaction, where glucose is oxidized by GOx, resulting in the production of H_2_O_2_ and gluconic acid. In this experiment, we utilized the affinity partitioning properties to encapsulate GOx within the protocell's inner phase, while maintaining consistent glucose concentration in the outer phase. As shown in Figure S4 (Supporting Information), the concentration of generated H_2_O_2_ increased in the presence of both GOx‐loaded protocells and GOx‐contained PBS solution, showing a dependence on glucose concentration. In contrast, negligible H_2_O_2_ was produced in the absence of GOx. The reaction rate plotted against the molar concentration of glucose exhibited typical Michaelis‐Menten behavior in both bulk solution and protocell solution (Figure 4E). We determined the *V*_max_ and *K*_m_ values by fitting the Michaelis‐Menten plots. *V*_max_ means the maximum reaction rate, and *K*_m_ reflects the binding affinity, with a lower value of *K*_m_ indicating a stronger affinity between the enzyme and the substrate. The estimated *V*_max_ for GOx loaded within protocells and without protocells is 504.73 and 625.86 µM min^−1^, respectively. The *K*_m_ values for GOx loaded within protocells and without protocells are 1.84 × 10^−3^ and 2.19 × 10^−3^ m, respectively. From the Michaelis‐Menten plots, we derived a catalytic rate constant *k*_cat_ of 1502.6 s^−1^ for GOx in bulk PBS solution and 1211.3 s^−1^ for GOx encapsulated within the protocells, suggesting a slightly inhibited catalytic function of GOx inside the coacervates. The decrease in *k*_cat_ may indicate a reduction in the activity of the GOx within the protocell. To visualize the spatial localization of H_2_O_2_, fluorescence microscopic images of a single GOx‐loaded protocell prepared with the presence of Amplex red were examined. Upon adding 10 × 10^−3^ m glucose, H_2_O_2_ was generated and subsequently oxidized Amplex red, leading to a sharp increase in red fluorescence only within the protocell interior, indicating the generation and retention of resorufin (Figure 4F,G and Figure S5, Supporting Information).

In the second step, the oxidation of glucose produced an acidic environment that facilitated the dissociation of Cu NDs, leading to the release of Cu^2+^ ions, which underwent a Fenton‐type reaction with H_2_O_2_ and generated hydroxyl radicals.^[^ ^19^ ^]^ To investigate the maximum efficiency of ROS generation by the protocells, we loaded different concentrations of Cu NDs within the protocells and tested the generation of ROS by adding a 3,3′,5,5′‐tetramethylbenzidine (TMB) solution (Figure S6, Supporting Information). Ultimately, a concentration of 20 µg mL^−1^ of Cu NDs was selected for subsequent experiments. In protocells with the same Cu NDs concentration, the total amount of generated ROS was directly determined by the concentration of glucose (Figure S7A,B, Supporting Information).

Since in vivo experiments require the long‐term stability of protocells under physiological conditions, we examined their structural stability by placing them in glucose solutions of varying concentrations and observing changes in turbidity over several days (Figure S8, Supporting Information). The results indicated that the protocells could maintain structural stability for at least one week.

### 2.4 Antibacterial Performance of the Coacervates

The coacervate protocells described in the study can consume glucose and generate ROS, making them effective in reducing glucose levels and killing bacteria. To evaluate their antibacterial properties, *Escherichia coli* (*E. coli*) and *Staphylococcus aureus* (*S. aureus*) were selected as bacterial models. After 24 h of coculturing with the bacterial solution, the group of protocells loaded with GOx and Cu NDs showed fewer bacterial colonies compared to the other groups, demonstrating excellent antibacterial effects against both gram‐negative and gram‐positive bacteria (Figure 4H). Furthermore, it was demonstrated that the presence or absence of DPPC membranes on the protocells did not have a significant effect on the antibacterial efficacy, consistent with the results of the ROS production test (Figures S9 and S10, Supporting Information).

Moreover, bacterial biofilms can pose significant obstacles to drug penetration and effectiveness, leading to drug resistance. Therefore, the effect of protocells on biofilm destruction was also explored (Figure S11, Supporting Information and Figure 4I). Crystal violet staining results revealed that the protocells produced sufficient ROS to deteriorate the bacterial biofilm of *E. coli*. Compared with traditional antibiotic therapies, such ROS‐based therapy exhibited effective antibacterial properties while avoiding the negative effects associated with the overuse of antibiotics. The antibacterial results were further supported by corresponding live/dead staining images of *E. coli* and *S. aureus* after coculturing with the protocell solution for 24 h (Figure 4J).

### 2.5 In Vitro Biocompatibility Tests

Before testing the feasibility of using these protocells in wound healing, their in vitro hemocompatibility and biocompatibility were assessed. For the hemocompatibility test, coacervate microdroplets and DPPC membrane‐coated coacervate microdroplets were separately incubated with red blood cells (RBCs). The results showed that membrane‐free microdroplets exhibited significantly higher hemolytic activity compared to membrane‐coated ones (Figure S12, Supporting Information). The hemolysis rate increased rapidly with increasing concentrations of uncoated coacervate microdroplets, reaching values over 5% at coacervate concentrations above 1 mg mL^−1^. In contrast, RBCs incubated with membrane‐coated coacervate microdroplets showed lower hemolytic activity (1.43 ± 0.28%) under 1 mg mL^−1^.

Cytotoxicity tests were then conducted by coculturing with 3T3 cells. CCK‐8 test results indicated that the DPPC‐coated coacervate microdroplets exhibited better cellular compatibility compared to membrane‐free ones (Figure 4K). Additionally, calcein‐AM staining images showed that 3T3 cells cocultured with the protocells exhibited good growth over 3 d (Figure S13A,B, Supporting Information). The improved hemocompatibility and cellular compatibility of DPPC membrane‐coated coacervates can be attributed to the blocking of detrimental contact interactions between living cells and the coacervate matrix. These results support their potential use for the delivery of therapeutic agents.

### 2.6 In Vivo Infected Diabetic Wound Healing Experiments

**Figure 5:**
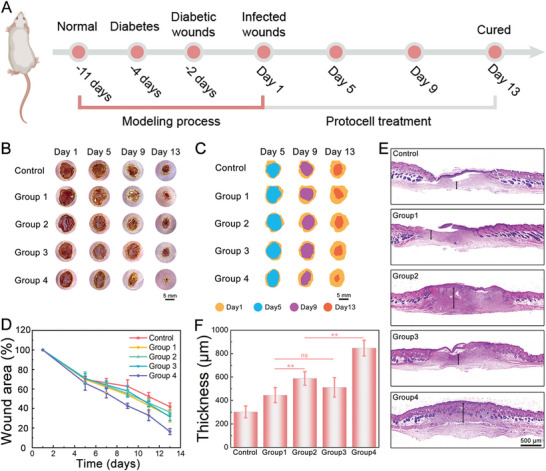
Evaluation of the therapeutic performance of the protocells on diabetic wound treatment. A) Timeline diagram of the different time points in animal experiments. B) Photographs of the wound states of mice in different groups within 13 d. C) Diagram of relative changes in the wound area corresponding to B. D) Measured changes in wound area of different groups. Data are shown as mean ± SD (*n* = 5). One‐way ANOVA and Tukey's test were performed. E) H&E staining images of skin tissues from different groups. F) Measured thickness of the granulation tissue on Day 13. Data are shown as mean ± SD (*n* = 5). One‐way ANOVA and Tukey's test were performed.

Having validated the above‐described properties of the coacervate‐based protocells, we explored their application in promoting diabetic wound healing. Considering that protocells with uniform size possess unified cascade catalytic reaction activity and thus antibacterial efficacy, in this section, microfluidic‐derived protocells were used in a wound healing assay. Mice with type I diabetes were selected and randomly divided into five groups, each with an 8 mm round wound on their back. The mice in the control group were treated with PBS buffer to soak the wounds. The remaining four groups received different treatments, including free protocells in Group 1, GOx‐loaded protocells in Group 2, Cu NDs‐loaded protocells in Group 3, and GOx+Cu NDs‐loaded protocells in Group 4. The condition of the wounds in each group was monitored and recorded with time, as depicted in **Figure** **5**A,B. The results showed that Group 4 exhibited superior wound closure compared to the other groups, indicating the most effective treatment effect. This observation was further supported by the quantification of the wound closure area, as shown in Figure 5C. Additionally, hematoxylin‐eosin (H&E) staining was performed to examine the states of the wound tissues. As depicted in Figure 5D,E, the wounds in Group 4 exhibited the thickest granulation tissue compared to the other groups, with the control group being the thinnest. The findings suggested that the application of protocells loaded with both GOx and Cu NDs resulted in significant wound healing improvements, offering a promising therapeutic approach for diabetic wound treatment.

**Figure 6:**
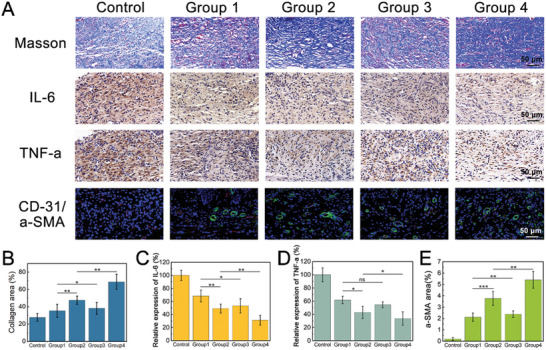
Biological mechanisms of wound healing by using the protocells. A) Masson staining, immunohistochemical staining of TNF‐𝛼 and IL‐6 expression, and immunofluorescence staining of 𝛼SMA and CD31 of wound area in different groups at Day 13. B) Statistic analysis of the collagen deposition area. Data are shown as mean ± SD (*n* = 5). One‐way ANOVA and Tukey's test were performed. C) The relative expression of IL‐6. Data are shown as mean ± SD (*n* = 5). One‐way ANOVA and Tukey's test were performed. D) The relative expression of TNF‐𝛼. Data are shown as mean ± SD (*n* = 5). One‐way ANOVA and Tukey's test were performed. E) Statistic analysis of α‐SMA area. Data are shown as mean ± SD (*n* = 5). One‐way ANOVA and Tukey's test were performed.

Additionally, Masson staining results demonstrated that collagens in Group 4 displayed a higher degree of directional alignment, indicative of an improved wound microenvironment and healing status (**Figure** **6**A,B). IL‐6 and TNF‐𝛼 are important indicators for assessing the inflammatory status. Compared to the other groups, Group 4 showed the least expression of these factors, which can be attributed to the distinctive anti‐bacterial effect of the GOx and Cu NDs‐loaded protocells (Figure 6C,D). Moreover, blood vessel formation is crucial for tissue restoration. Immunofluorescent staining results indicated that Group 4 exhibited the greatest vessel density (Figure 6E). Taken together, these results demonstrated that the application of the glucose‐responsive protocells is valuable in diabetic wound management.

## 3 Conclusion

In this study, we introduced a type of cascade catalytic protocells loaded with GOx and Cu NDs for use in diabetic wound healing. An active microfluidic approach was employed to generate GOx+Cu ND‐encapsulated DEAE‐dextran/dsDNA coacervate microdroplets of uniform size, and a spontaneous self‐assembly process yielded a DPPC membrane at the droplet surface, which improved biocompatibility. When applied to the diabetic wound site, membrane‐bound coacervate droplets, that is, protocells, took advantage of the hyperglycemic environment to generate H_2_O_2_ through GOx catalysis. Simultaneously, the locally acidic environment triggered the decomposition of Cu NDs within the protocells, generating Cu^2+^, which further catalyzed H_2_O_2_ to produce antibacterial ROS. The effective antibacterial performance of the protocells was demonstrated both in vitro and in vivo, and their wound‐healing performance in diabetic mice was validated. These results highlight the ability of coacervates to accommodate cascade reactions and demonstrate that coacervate‐based protocells can serve as microcarriers for therapeutic agents. We believe that coacervate‐based smart materials could play a role in the construction of stimuli‐responsive vehicles, and hold tremendous promise for drug delivery and disease treatment.

## 4 Experimental Section

### Materials

Diethyl aminoethyl‐dextran hydrochloride (DEAE‐dextran, *M*_w_ 500 kDa), deoxyribonucleic acid (DNA, low molecular weight, from salmon sperm), glucose, 3,3′,5,5′‐tetramethylbenzidine (TMB), and glucose oxidase (GOx) were purchased from Sigma. Fluorescein 5‐isothiocyanate (FITC) and streptozocin (STZ) were purchased from Solarbio. Polyvinylpyrrolidone (PVP, *M*_w_ 10 kDa), rhodamine B isothiocyanate (RBITC), 1,2‐dipalmitoyl‐sn‐glycero‐3‐phosphocholine (DPPC), sodium hydroxide (NaOH), and chloride dihydrate (CuCl_2_·2H_2_O) were purchased from Aladdin. Cell Counting Kit‐8 (CCK‐8) Kit, Hydrogen Peroxide Assay Kit, Calcein/PI Live/Dead Viability/Cytotoxicity Assay Kit, and Calcein AM were purchased from Beyotime. Hydrogen peroxide (H_2_O_2_, 30%), acetone, and methanol were purchased from Sinopharm Chemical Reagent Co., Ltd.

### Preparation of Cu NDs

Cu NDs were synthesized following a previously reported procedure.^[^ ^19^ ^]^ In summary, 500 mg PVP was dissolved in a CuCl_2_·2H_2_O solution (5 mL, 0.01 m). Subsequently, 100 µL of NaOH (5 mL, 0.02 m) and H_2_O_2_ (30%) solutions were sequentially added to the aforementioned mixture. After 30 min of stirring, the Cu NDs were collected and washed with water multiple times. The nanodots were characterized using high‐resolution transmission electron microscopy.

### Synthesis of Fluorescence‐Labeled GOx and Cu NDs

GOx was fluorescently labeled with FITC and RBITC using a previously reported method.^[^ ^10^ ^]^ In summary, an aqueous solution containing 50 × 10^−12^ m GOx and 500 × 10^−12^ m FITC was incubated at room temperature for 4 h, followed by 6 h of constant stirring (4 °C, 500 rpm). Excess dye was eliminated by dialysis against a PBS solution at 4 °C for 12 h. The synthesis process for RBITC‐labeled GOx followed the same protocol.

The FITC‐labeled Cu NDs were generated by replacing PVP with FITC‐labeled PVP.^[^ ^23^ ^]^ To synthesize FITC‐PVP, 1 mL of 2 mg mL^−1^ FITC in a 0.1 m sodium carbonate/bicarbonate buffer was added to 10 mL of 25 mg mL^−1^ PVP in the same buffer. The mixture was incubated away from light for 3 h at room temperature. The FITC‐PVP was then purified and washed with acetone before being collected through freeze‐drying. The subsequent synthesis procedure for FITC‐labeled Cu NDs was the same as for the regular Cu NDs.

### Determination of Partition Constant for GOx

The partition constant (*K*) for GOx in the coacervate solution was determined from the ratio of concentrations in the coacervate phase (*C*_in_) and continuous aqueous phase (*C*_out_), and given by *K* = *C*_in_/*C*_out_. After capturing GOx into the coacervate phases, the bulk coacervate phase was separated from the coacervate solution by centrifugation (5000 rpm, 20 min). The concentration of the GOx in the upper aqueous phase was monitored by detecting adsorption at 455 nm using UV–vis spectroscopy while that in the dense coacervate phase was determined after disassembly of the coacervate phase using 1 m NaCl solution.

### Preparation of DPPC Membrane‐Coated GOx/Cu NDs‐Containing Protocells by Mixing

Aqueous suspensions of coacervate microdroplets with a positive surface potential were prepared by mixing 400 µL of DEAE‐dextran (10 mg mL^−1^) and 200 µL of dsDNA (10 mg mL^−1^). The turbidity of the coacervate solutions was estimated by measuring the absorbance at 600 nm via a UV spectrophotometer. For the preparation of GOx/Cu NDs‐containing coacervates, 10 µL of a 2 mg mL^−1^ GOx solution and 20 µL of a Cu NDs solution were pre‐mixed with the DEAE‐dextran solution before further mixing with the dsDNA solution. To prepare DPPC membrane‐encapsulated coacervate protocells, 15 µL of a 20 mg mL^−1^ DPPC ethanol solution was added to 600 µL of a DNA/DEAE‐dextran coacervate microdroplet suspension (10 mg mL^−1^). The mixture was then incubated for 10 h at 4 °C.

### Generation of Coacervate Microdroplets by Microfluidics

A typical droplet generation experiment is operated as follows. First, 10 mg mL^−1^ DEAE‐dextran in PBS buffer and 10 mg mL^−1^ dsDNA in PBS buffer were well mixed with a volume ratio of 2:1. Then, the bulk DEAE‐dextran/dsDNA coacervate phase was separated from the coacervate‐containing solution by centrifugation (5000 rpm, 20 min) and removing the supernatant. The collected coacervate phase flowed into the injection tube of the microfluidic device via a syringe. PBS buffer served as the outer phase fluid and flowed into the collection tube. A typical set of flow rates of the inner and outer fluid was 20 µL h^−1^ and 5 mL h^−1^, respectively. Sinusoidal perturbation was applied on the inner phase with the use of a mechanical vibrator for active droplet generation. For each type of coacervate droplets generated from microfluidics, the DPPC membrane coating procedure was the same: 20 mg mL^−1^ DPPC ethanol solution was diluted by PBS to achieve a final concentration of 0.5 mg mL^−1^, which served as the outer phase. After the generation, the protocells were collected and further incubated at 4 °C for 12 h.

### Detection of GOx Enzymatic Activities and H_2_O_2_ Generation

A GOx‐loaded protocell solution, a free protocell solution, and a bulk PBS solution of GOx were added to different concentrations (0 × 10^−3^, 1 × 10^−3^, 2 × 10^−3^, 3 × 10^−3^, 4 × 10^−3^, 5 × 10^−3^, 10 × 10^−3^, 15 × 10^−3^, and 20 × 10^−3^ m) of glucose solutions. After incubating for 3 h, the solutions were centrifuged at 5000 rpm for 10 min to avoid the disturbance of turbidity in protocell‐containing solutions on the observation results. The supernatant was then collected, and the generation of H_2_O_2_ was detected using an H_2_O_2_ assay kit. To visualize the generation of H_2_O_2_ within the protocells, 2 × 10^−6^ m of Amplex red was added to the solution containing GOx‐loaded protocells. Subsequently, 5 × 10^−3^ m glucose was added to trigger the GOx reaction. In the reaction, GOx converts glucose into gluconic acid and H_2_O_2_, and the generated H_2_O_2_ oxidizes Amplex red to resorufin, resulting in red fluorescence.

### Detection of ROS Generation

A group of GOx+Cu NDs‐loaded protocell solutions of the same concentration were mixed with different concentrations (0 × 10^−3^, 1 × 10^−3^, 2 × 10^−3^, 3 × 10^−3^, 4 × 10^−3^, 5 × 10^−3^, 10 × 10^−3^, 15 × 10^−3^, and 20 × 10^−3^ m) of glucose solutions, respectively. After mixing, the combined solution was left to react for 5 h. Then, 100 × 10^−3^ m TMB was added, and the mixture was equilibrated for 30 min. Finally, the solutions were centrifuged at 5000 rpm for 10 min, and the supernatant was collected. The oxidized TMB absorbance at 650 nm was measured to quantify the production of ROS.

### In Vitro Antibacterial Assay

Bacterial models of *E. coli* and *S. aureus* were employed. Initially, the bacterial suspension was centrifuged at 2000 rpm for 20 min. The resulting precipitate was then resuspended in PBS buffer until the turbidity value reached 0.4. Subsequently, 5 × 10^−3^ m glucose was added to the suspension. The experiment was divided into the control, free protocells, GOx‐loaded protocells, and GOx+Cu NDs‐loaded protocells groups. In each group, 200 µL of the bacterial resuspension was thoroughly mixed with 50 µL of the respective treatment solution. After incubating for 24 h, the solution was pipetted for plate culture. To visualize the bacterial death rate, the solution from each of the four groups was washed with 1 m NaCl solution. The bacteria were then resuspended in PBS and stained using a live/dead staining kit. The stained bacterial suspensions were observed under a confocal microscope to assess the viability of the bacteria.

### In Vitro Antibiofilm Study

Initially, 100 µL of *E. coli* suspension was added to each well of a 96‐well plate. The bacterial suspensions were incubated for 48 hours to allow biofilm formation. Subsequently, the samples received four types of treatments: PBS, free protocell solution, GOx‐loaded protocell solution, and GOx+Cu NDs‐loaded protocell solution. After coculturing with 100 µL of the respective treatment solution for 24 h, the supernatant was removed, and the precipitates were mildly washed with 1 m NaCl solution, followed by a PBS wash. The biofilm was fixed with 100 µL of methanol for 15 min, after which crystal violet dye (200 µL, 0.2%) was added and incubated for 30 min. Excess dye was then removed, and the mixture solutions were washed with PBS. Subsequently, 200 µL of 33% acetic acid was added to each well and incubated for 30 minutes. Then, the absorbance at 590 nm was measured using a UV spectrophotometer.

### Hemolysis Assays

The hemolytic activity of the coacervate microdroplets before and after coating with DPPC was measured by colorimetric analysis. In brief, 100 µL of mouse red blood cells in PBS (20% v/v) were incubated with 300 µL of different concentrations of coacervate microdroplets or protocells solution (0.01, 0.025, 0.05, 0.075, 0.1, 0.25, 0.5, 0.75, and 1 mg mL^−1^ in PBS buffer) for 90 min. Subsequently, the samples were centrifuged at 2000 rpm for 20 min. Hemolysis rate was estimated by measuring the absorption at 540 nm.

### In Vitro Biocompatibility Test

The cytotoxicity of the protocells on 3T3 cells was assessed using the CCK‐8 assay. 96‐well plates were used to incubate 3T3 cells for 24 h. Next, the medium was replaced with DMEM‐containing coacervate microdroplets with or without a DPPC membrane at concentrations of 0.001, 0.01, 0.05, 0.1, 0.5, and 1 mg mL^−1^. DMEM medium without coacervate microdroplets was the control. After incubation for 12 h, a CCK‐8‐containing medium (10 µL CCK‐8+100 µL fresh DMEM) was used for further incubation for 1.5 h. For the cell viability test, 3T3 cells were cultured with 0.05 mg mL^−1^ protocells and an equal volume of PBS in DMEM for 1, 2, and 3 d. The cells were then stained with 100 µL of calcein‐AM.

### Wound Healing Assay

Animal experiment protocols were reviewed and approved by the institutional animal care committee in line with the National Ministry of Health. All animal experiments in this study were approved by Fudan University (202308026Z).

Female BALB/c mice, aged 4–5 weeks, were obtained from Suzhou Xishan Biotechnology Inc. and maintained under SPF conditions. Streptozotocin (10 mg mL^−1^, in 0.1 m citrate buffer) was intraperitoneally injected into the mice (200 mg kg^−1^) after 12 h fasting. The mice were then fed with normal food, and their blood glucose levels were monitored after the injection. Mice with over 11.1 × 10^−3^ m blood glucose level were considered to have developed a diabetic model.

Next, 8 mm round wounds were created on the back of the mice. Subsequently, 10 µL of an *S. aureus* suspension (1.3 × 10^8^ CFU mL^−1^) was applied to the wounds. After 2 d, diabetic mice with festering wounds were considered to have developed an infected diabetic wound model.^[^ ^24^ ^]^ This day was considered as the first day of the wound‐healing experiment. In the control group, the wound was treated with 20 µL of PBS. Mice in the other four groups were treated with equal volumes of protocells (Group 1), GOx‐loaded protocells (Group 2), Cu NDs‐loaded protocells (Group 3), and GOx+Cu NDs‐loaded protocells (Group 4), respectively. Wound images were recorded on Days 1, 5, 9, and 13. On the last day of treatment, all mice were euthanized and the granulation tissues were properly processed for further analysis.

### Statistical Analysis

Results are presented as mean ± standard deviation (SD). All presented results represent at least three independent experiments. The number of samples (*n*) for each panel was described in detail. Student's t‐test was used for comparisons between the two groups and multiple comparisons were performed using one‐way analysis of variance (ANOVA), followed by Tukey's test. Statistical significance was defined as ns: no significant, * *p* < 0.05, ** *p* < 0.01, *** *p* < 0.001 in the figures.

## Conflict of Interest

The authors declare no conflict of interest.

## Supporting information

Supporting Information

## Data Availability

The data that support the findings of this study are available from the corresponding author upon reasonable request.
