# Supplementary material for: Glucose Responsive Coacervate Protocells from Microfluidics for Diabetic Wound Healing
Source: Adv Sci (Weinh). 2024 May 20;11(28):2400712. doi: 10.1002/advs.202400712 (PMC11267285; doi:10.1002/advs.202400712)
Supplement: Supplementary file 1 — Supporting Information [file ADVS-11-2400712-s001.docx]

Supporting information

**Glucose Responsive Coacervate Protocells from Microfluidics for Diabetic Wound Healing**

*Chong Wang^1^, Xinyuan Yang^1^, Qiao Wang^1^, Linyi Zhang^1^, Luoran Shang^1*^*

1 Shanghai Xuhui Central Hospital, Zhongshan-Xuhui Hospital, and the Shanghai Key Laboratory of Medical Epigenetics, the International Co-laboratory of Medical Epigenetics and Metabolism (Ministry of Science and Technology), Institutes of Biomedical Sciences, Fudan University, Shanghai, China

Email: luoranshang@fudan.edu.cn


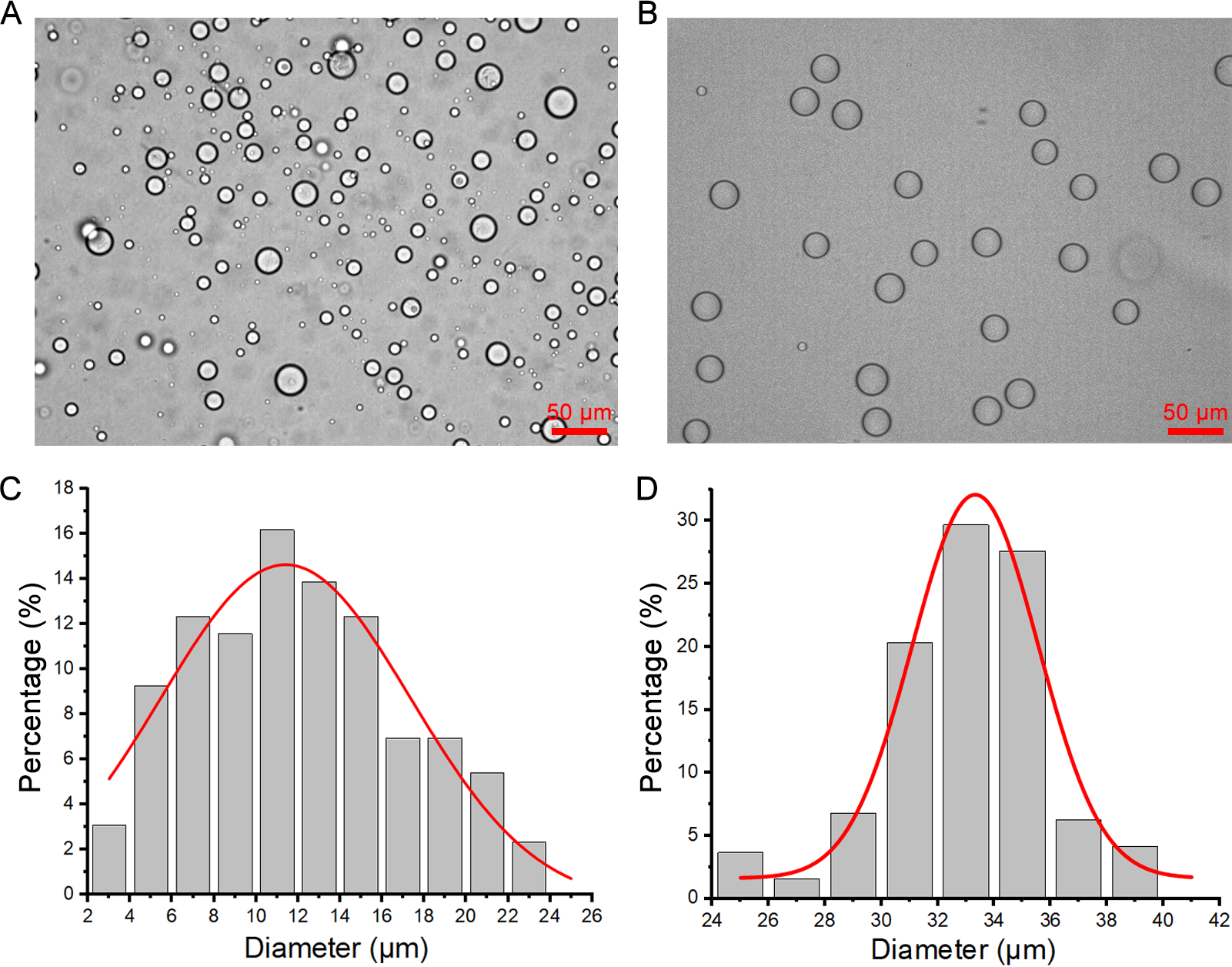


**Figure S1**. **Coacervate microdroplets generated through different methods**. **A**. Microscopic image of coacervate microdroplets generated by directly mixing DEAE-dextran and dsDNA solution. **B**. Microscopic image of coacervate microdroplets generated by the microfluidic approach. **C**. Frequency distribution histogram illustrating the size distribution of coacervate microdroplets obtained through direct mixing (n=100). **D**. Frequency distribution histogram depicting the size distribution of coacervate microdroplets generated via microfluidics (n=100). The photocells generated through microfluidics exhibit a lower relative standard deviation (0.087) compared to those produced through direct mixing (0.411), demonstrating superior uniformity.


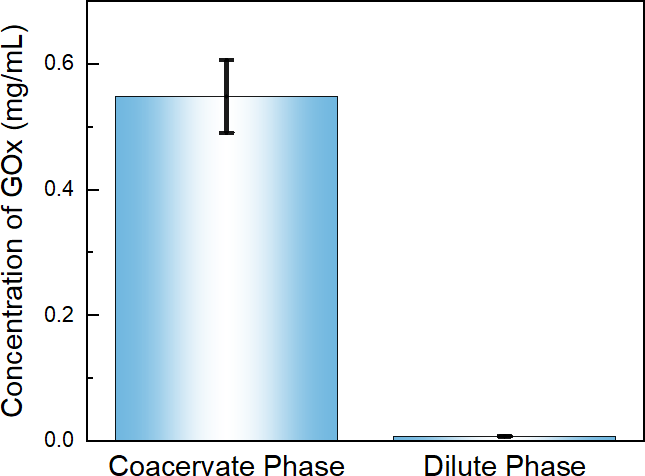


**Figure S2**. Concentrations of GOx inside and outside the coacervate phase, respectively. Data are shown as mean ± SD (n = 5).


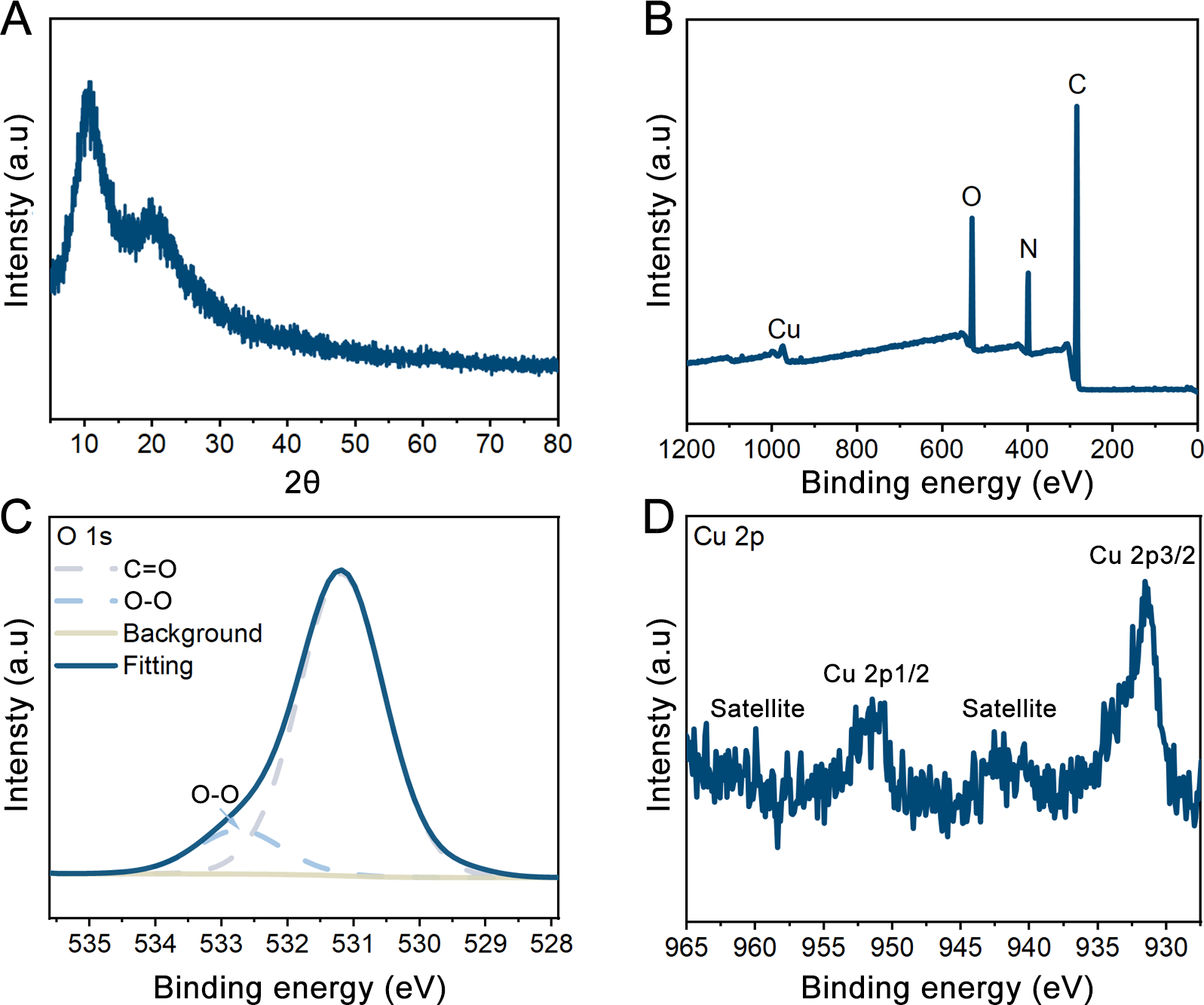


**Figure S3. XRD and XPS spectra of Cu NDs.** **A**. XRD spectra of Cu NDs. **B**. High-resolution survey XPS spectra of Cu NDs. **C**. O 1s XPS spectra of Cu NDs. **D**. Cu 2p XPS spectra of Cu NDs.


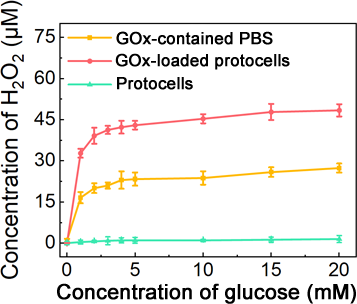


**Figure S4**. Production of H_2_O_2_ under different conditions. Data are shown as mean ± SD (n = 5).


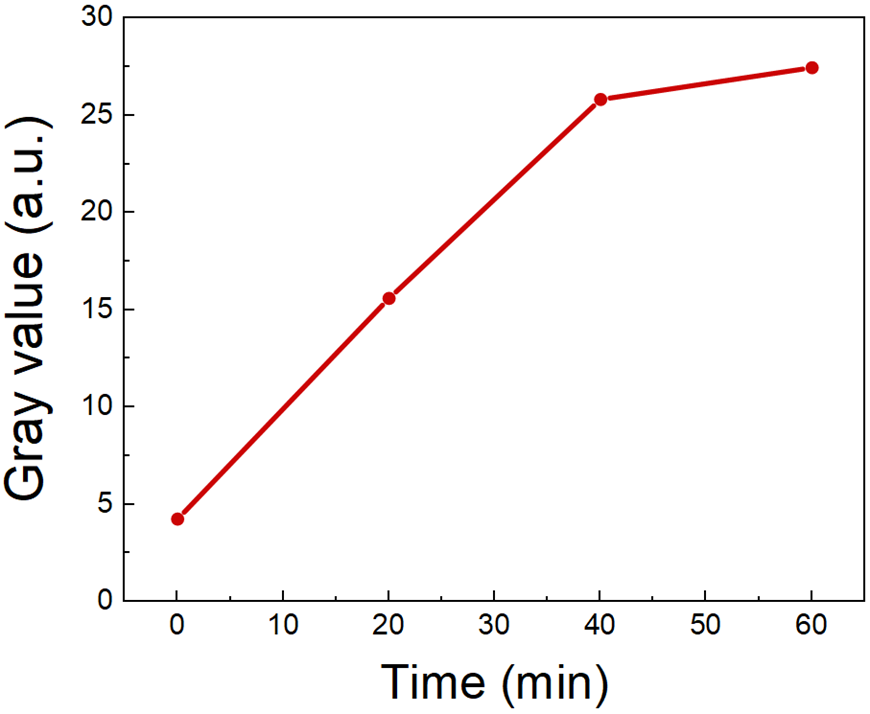


**Figure S5**. Plot of changes in mean fluorescence recorded inside the protocells (corresponding to Figure 4F).


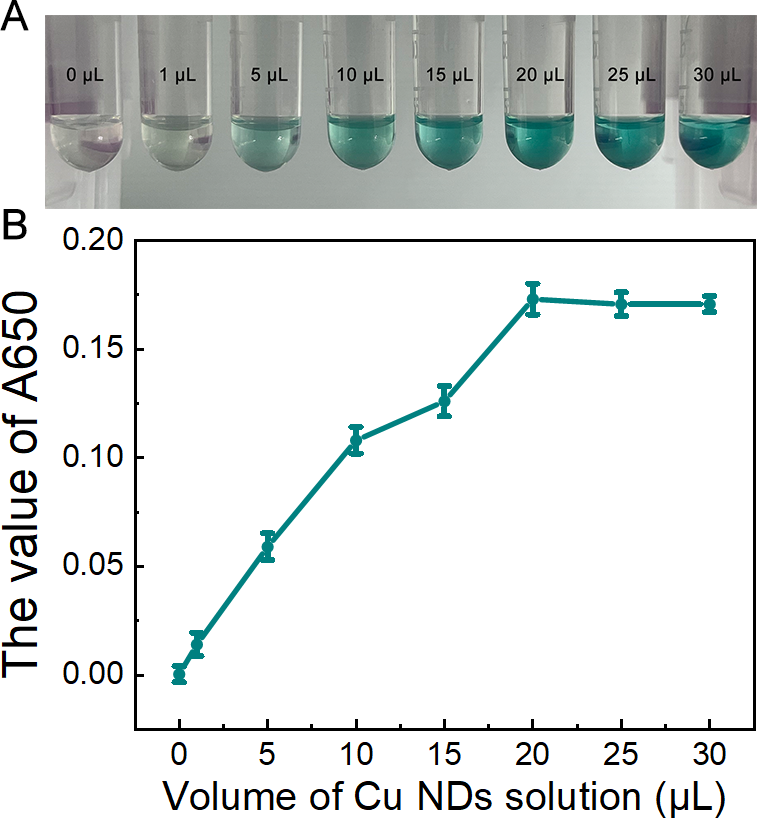


**Figure S6.** **Generation of** **oxidized TMB (ox-TMB) with protocells loaded with different amounts of Cu NDs**. **A**. Photographs of the ox-TMB supernatant displaying the generation of ROS by GOx+Cu NDs-loaded protocells with varying amounts of Cu NDs (0, 1, 5, 10, 15, 20, 25, 30 μL, respectively). **B**. Plot of the generation of ox-TMB by GOx+Cu NDs-loaded protocells with varying amounts of Cu NDs (0, 1, 5, 10, 15, 20, 25, and 30 μL, respectively). Data are shown as mean ± SD (n = 5).


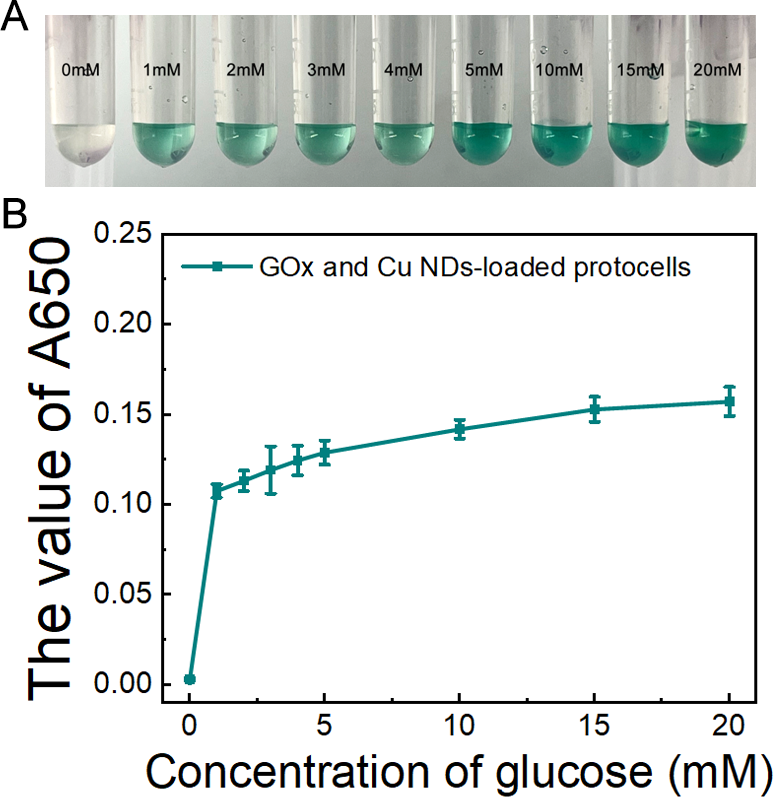


**Figure S7**. **Generation of oxidized TMB (ox-TMB) with protocells with different concentrations of glucose**. **A**. The supernatant from the solution of GOx+Cu NDs-loaded protocells after reacting with varying concentrations of glucose (depicted in each image). The identification of reactive oxygen species (ROS) generation was facilitated using TMB as an indicator. **B**. Changes in the absorbance of oxidized TMB at 650 nm as a function of glucose concentration for GOx/Cu NDs cascade reaction in protocells. Data are shown as mean ± SD (n = 5).


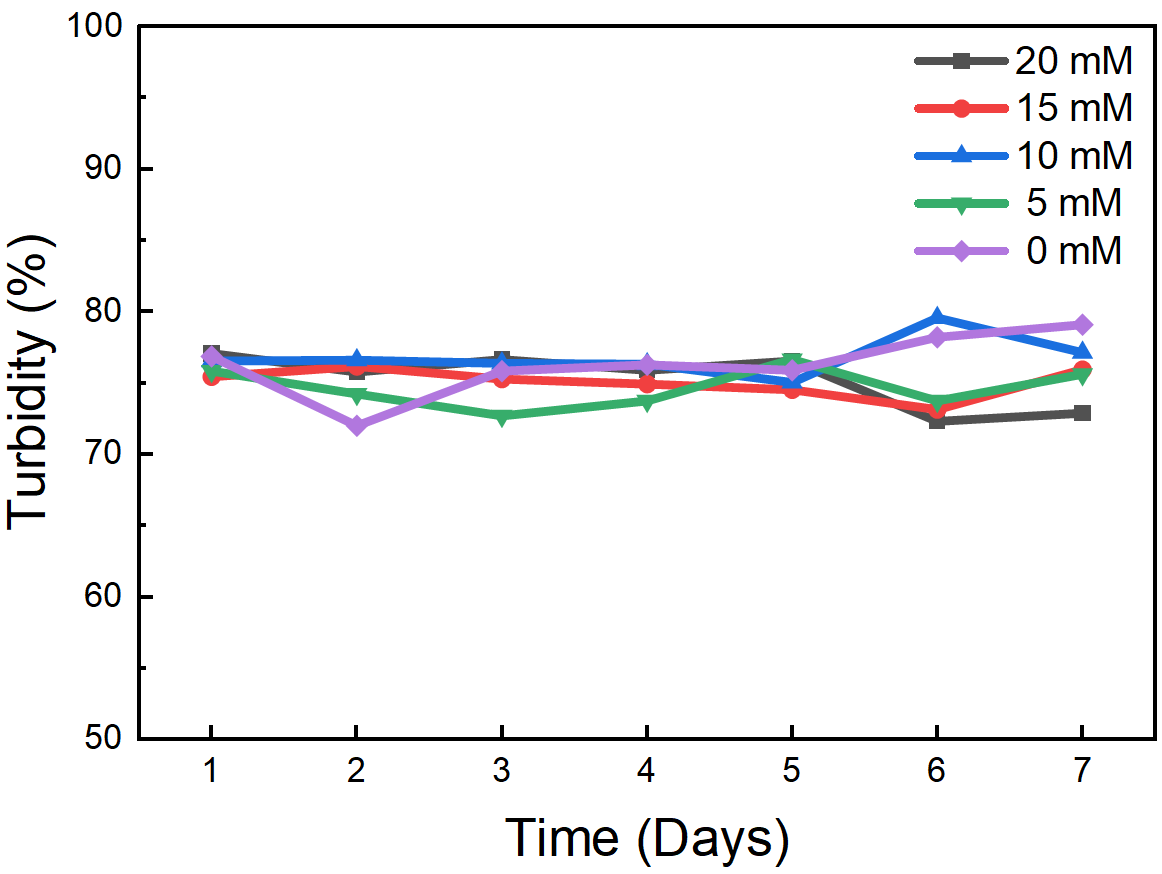


**Figure S8**. The plot of turbidity changes over time in protocell solutions containing different concentrations of glucose. Data are shown as mean ± SD (n = 5).


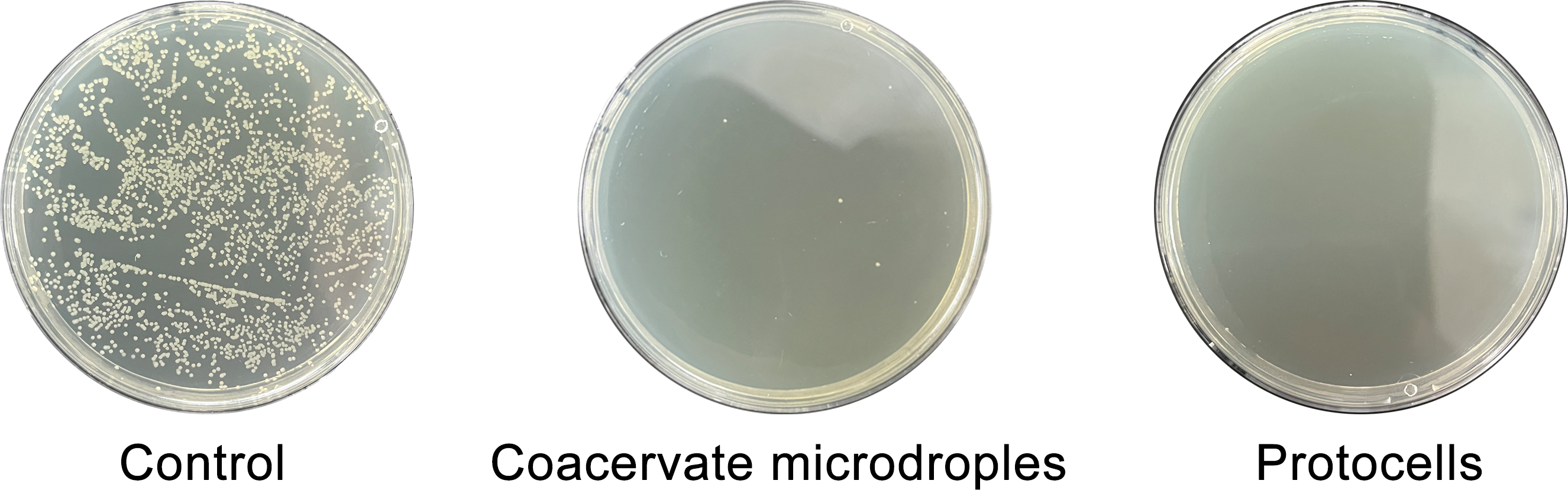


**Figure S9**. Photographs of *E. coli* bacterial colonies on the culture plate after being treated with GOx+Cu NDs-loaded coacervate microdroplets with or without DPPC membrane coating.


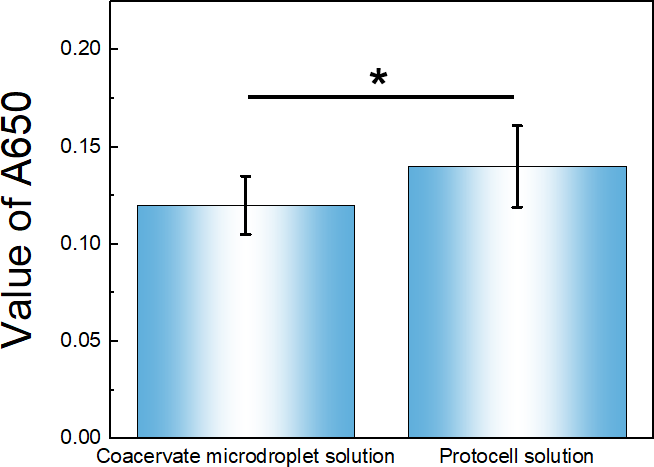


**Figure S10**. Statistic analysis of ox-TMB generation in GOx+Cu NDs-loaded coacervate microdroplet solution and GOx+Cu NDs-loaded protocell solution. Data are shown as mean ± SD (n = 5). Two-tailed unpaired Student's t-test was performed for the comparisons between the two groups.


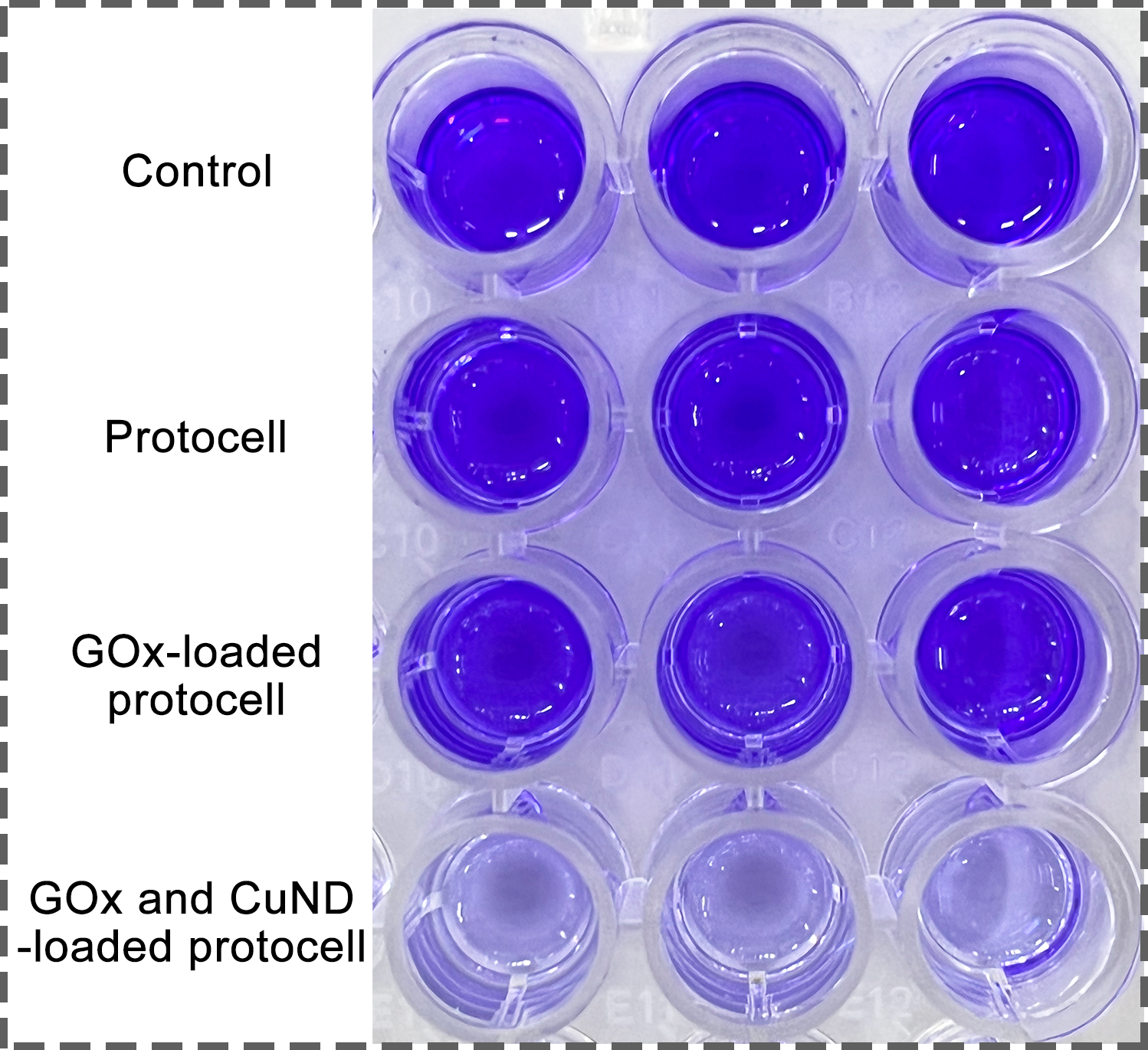


**Figure S11**. Images of antibiofilm performance of different treatments.


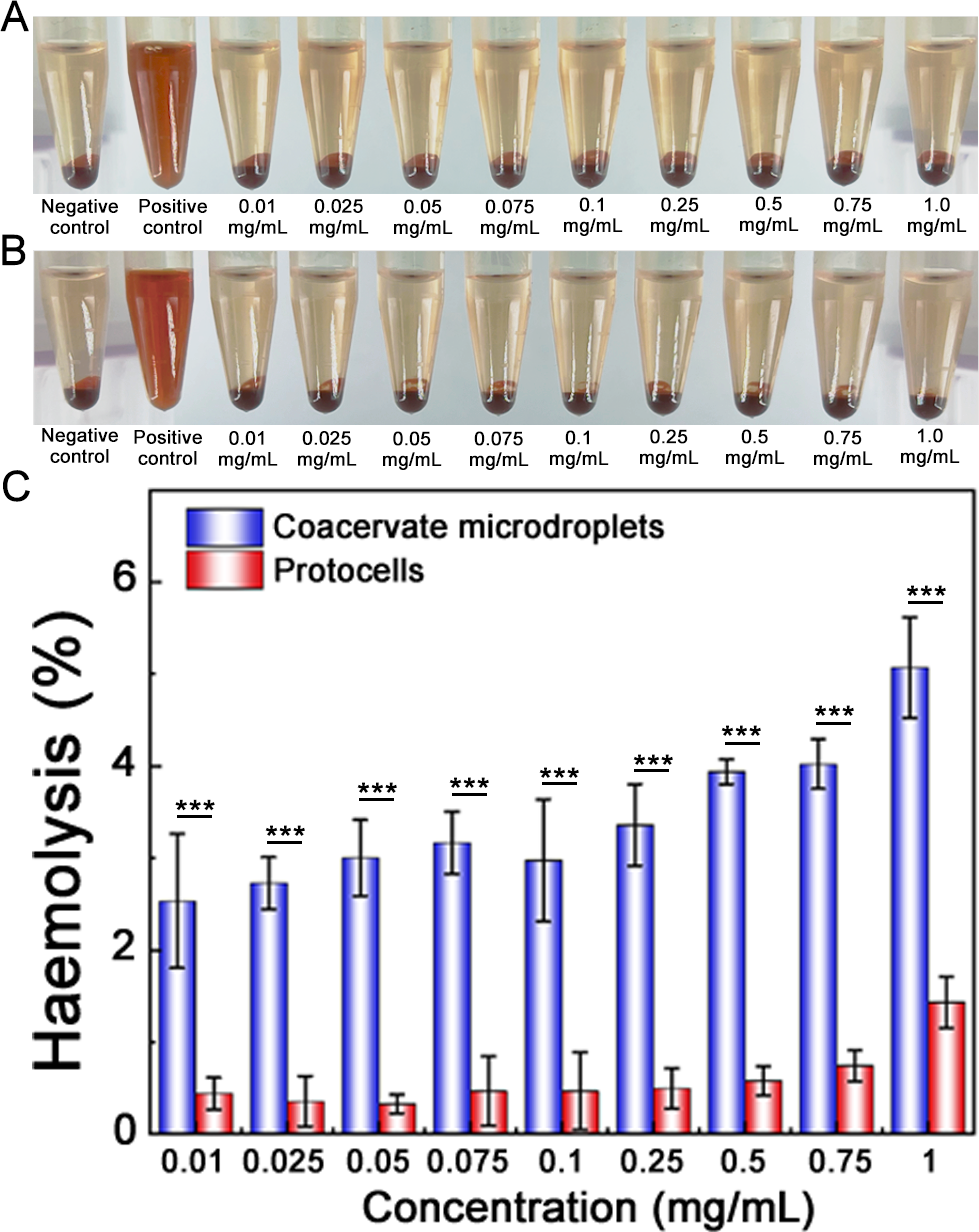


**Figure S12**. **Hemolysis assay results**. **A**. Photographs of the centrifuged suspension of red blood cells (RBCs) after incubation with different concentrations of coacervate microdroplets. **B**. Photographs of the centrifuged suspension of RBCs after incubation with different concentrations of protocells. **C**. Plot of the hemolysis rate against the concentration of uncoated and DPPC membrane-coated coacervate droplets. Data are shown as mean ± SD (n = 5). Two-tailed unpaired Student's t-test was performed for the comparisons between the two groups.


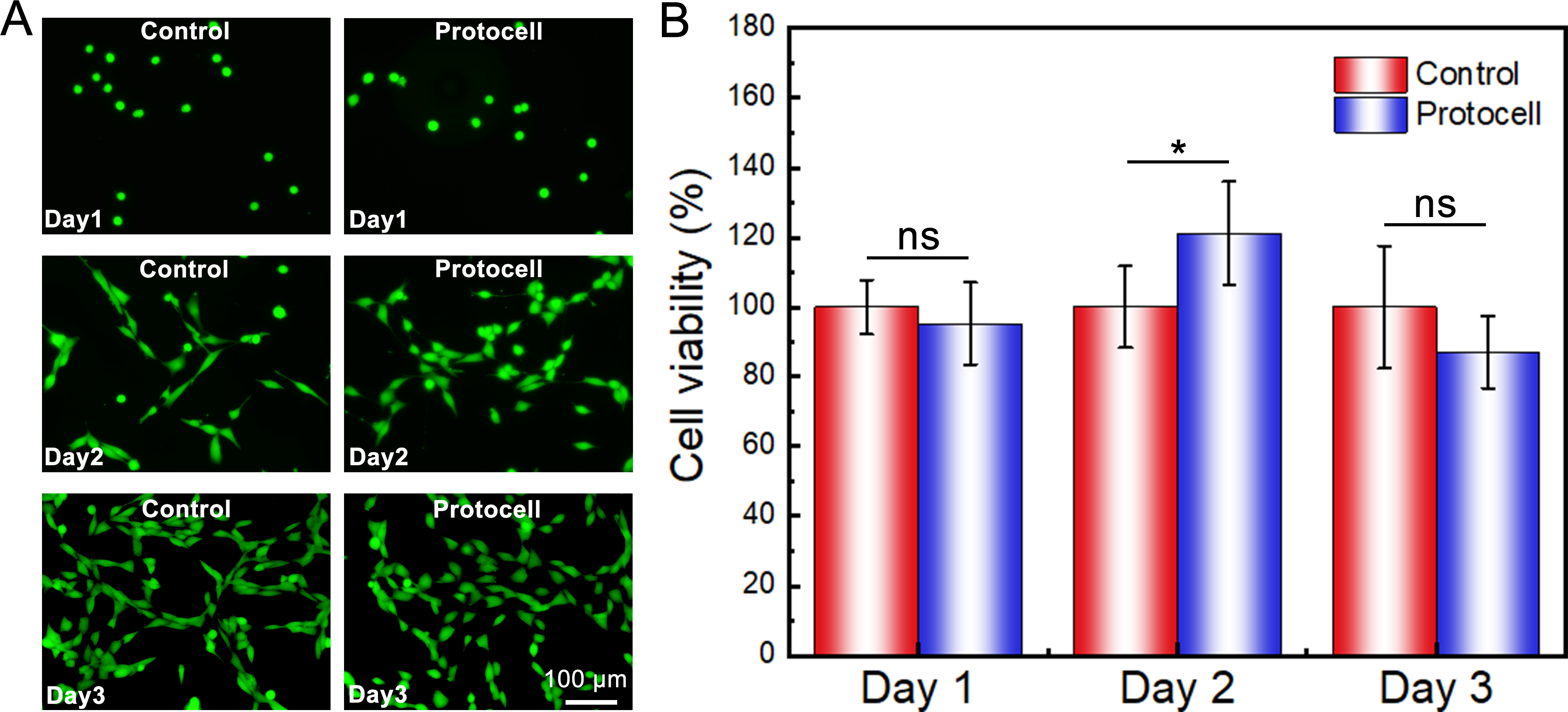


**Figure S13**. **Cell viability test results**. **A**. Fluorescence images of 3T3 cells co-cultured with the protocells on day 1, 2, and 3, stained with the calcein-AM kit. **B**. The cell viability of 3T3 cells, co-cultured with either PBS solution (control) or protocells, assessed using the calcein-AM kit. Data are shown as mean ± SD (n = 5). Two-tailed unpaired Student's t-test was performed for the comparisons between the two groups.
